# Treatment of the Atrophic Upper Jaw: Rehabilitation of Two Complex Cases

**DOI:** 10.1155/2013/154795

**Published:** 2013-07-28

**Authors:** Andrea Enrico Borgonovo, Andrea Marchetti, Virna Vavassori, Rachele Censi, Ramon Boninsegna, Dino Re

**Affiliations:** ^1^Istituto Stomatologico Italiano, Department of Oral Rehabilitation, School of Oral Surgery, University of Milan, via Pace, 21, 20122 Milan, Italy; ^2^Department of Implantology and Periodontology III, Istituto Stomatologico Italiano, Milan, Italy; ^3^Department of Clinical and Experimental Sciences, University of Brescia, Italy

## Abstract

In reconstructive surgery, the fresh frozen homologous bone (FFB) represents a valid alternative to the autologous bone, because FFB allows bone regeneration thanks to its osteoinductive and osteoconductive properties. The purpose of this work is to describe the surgical-implant-prosthetic treatment of two complex cases using FFB. In particular, fresh frozen homologous bone grafts were used to correct the severe atrophy of the maxilla, and, then, once the graft integration was obtained, implant therapy was performed and implants placed in native bone were immediately loaded.

## 1. Introduction

The implant-prosthetic rehabilitation is a current practice in clinic dentistry and is characterized by safe and predictable results in the long term [1]. However, in order to obtain the success of implant therapy, in the preliminary stages it is essential to assess and classify the amount of available bone. In fact, this evaluation is fundamental for the correct implant placement, according to the principles of modern prosthetically driven implant placement [2].

Several classifications have been proposed to assess the amount of available bone. In the Lekholm and Zarb [3] classification (1985), the jaw bone shape is classified on a five degree scale. Cawood and Howell [4] (1988) proposed another classification that differentiates the atrophies according to an analysis of three-dimensional alveolar ridges. The presence of unfavorable crestal anatomy, which may result from different situations such as atrophy, periodontal disease, iatrogenic or congenital defects, trauma, or oncological resection, is not an absolute contraindication to dental implant placement. In fact, with the advances and evolution occurring in implant dentistry, new surgical techniques have been developed and refined in order to allow the correction of bone defects and the implant-prosthetic management of compromised sites.

One of the most common procedures for the correction of bone defects involves autologous (or autogenous) bone grafting (bone is harvested from the patient's own body). Autologous bone is typically harvested from intraoral sources [5] as the chin, the mandibular ramous, the tuber maxilla or from extraoral sources as the iliac crest, the fibula, and even parts of the skull [6]. Other graft materials, which are used in clinical practice, are the xenograft bone substitutes, derived from a species other than human, such as bovine, the allograft bone, like autogenous bone which is derived from humans, and at last, the artificial bone, such as bioglass, hydroxyapatite, or calcium phosphate [2]. For the reconstruction of extended bone defects, autologous or homologous bone grafts are preferred, in form of blocks, in order to restore the correct vertical and/or horizontal dimensions.

Only recently, the homologous bone has been introduced in the reconstructive surgery and maxillofacial surgery, although it has been used for many years in orthopedics for complex surgical operations with clinical results consolidated in the long term [7, 8]. The homologous bone is obtained from a living donor (usually from patients undergoing total hip replacement) or from cadavers [9]. In the latter case, the harvesting is made within 12/24 hours of death, in a sterile environment. The parts that are collected are then sent to the Tissue Bank to be subjected to serological tests, tests to detect antibodies and antigens and blood culture. Once completed these exams, the bone is processed and depending on the type of preparation, three distinct physical forms can be distinguished.

- “Fresch frozen” (FFB)—the processing of the fresh frozen homologous bone does not provide decalcification nor irradiation. The FFB is initially disinfected with a polichemotherapic disinfection solution (72 hours at −4°C), then washed with saline solution, divided into blocks, packed in double sterile pouch, and preserved at −80°C in a tank [10];
- lyophilized homologous bone (FDB)—the bone tissue is ground into particles of 500 microns–5 mm, delipidated with pure ethanol, dehydrated, and frozen;
- demineralized and lyophilized homologous bone (DFDB)—in addition to the processes for freeze-drying, the bone is subjected to a further step that provides for the immersion in citric acid for 6–16 hours in order to demineralize the resulting particles.

Each of these types of homologous bone has precise indications, management, and contraindications [11, 12].

In implant surgery, the standard protocol for the management of complex cases in which bone grafting is performed, includes healing times of not less than 5-6 months for graft integration [13]. After this period and after verifying the success of the reconstructive therapy, dental implants can be placed in the grafted sites. The implant techniques, as described by Brånemark [14], require a submerged healing of endosseous implants placed in regenerated bone for a period of about 6 months and 3-4 months for implants placed in native bone [15]. At the end of this period, it is possible to functionalize the implants with temporary restorations. Although the protocol proposed by the Swedish school is still valid, the research has been directed towards the study of new protocols that present a shorter healing phase in order to reduce the duration of overall rehabilitation. In particular, considering the immediate loading techniques for dental implants [16, 17], several clinical studies [18, 19] showed that the immediate loading protocols can be applied with predictable results for implants placed in native bone, and excellent results were obtained also for implants placed in areas reconstructed with bone grafts but only in certain selected cases.

In this paper, the surgical-implant-prosthetic treatment of three complex cases is described. In particular, fresh frozen homologous bone grafts were used to correct the severe atrophy of the maxilla, and, then, once the graft integration was obtained, implant therapy was performed and implants placed in native bone were immediately loaded.

## 2. Case Series

**Figure 1:**
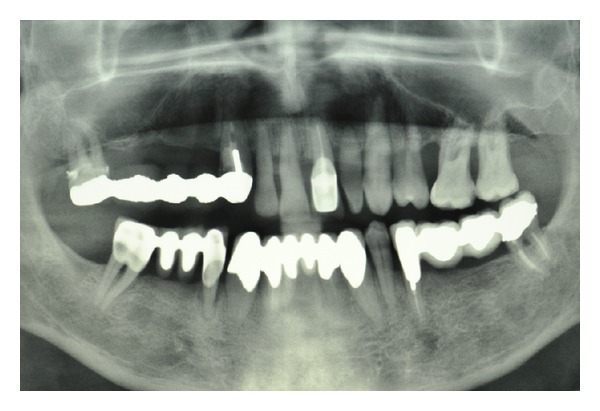
Case 1: Initial panoramic radiograph (OPT).

*Case 1*. The patient A. M., 56 years old, male came to our attention presenting, in the upper jaw, edentulous multiple sites, mobility of the remaining teeth, periodontal disease, and severe atrophy of the maxillary edentulous alveolar ridge. The patient clearly required that the prosthetic rehabilitation be exclusively fixed and did not accept provisional phases with removable dentures. Based on the clinical examination and the evaluation of radiographs (panoramic radiograph (Figure 1) and computed tomography of the maxilla with DentalScan reconstructions), it was decided to treat the atrophic upper jaw with a combined sinus lift procedure and local ridge augmentation using bone grafts in the areas from 1.4 to 1.6, performed under general anesthesia. In addition, it was decided to conserve not compromised teeth until the bone grafts were integrated in order to proceed, in a second phase, with implant placement and immediate loading.

The patient was adequately informed about the use of FFB grafts and the subsequent implant-prosthetic treatment plan. The preoperative phase included blood tests, an interview with the anesthesiologist for the general anesthesia, the signature of the informed consent form for the surgery, the test to determine the patient's blood group, and the signature of a specific informed consent form for the bone graft from the Tissue Bank. Once the preliminary phase was completed, the tissue specimen was then booked at the reference Tissue Bank, and in particular, a specimen harvested from the iliac crest was required.

**Figure 2:**
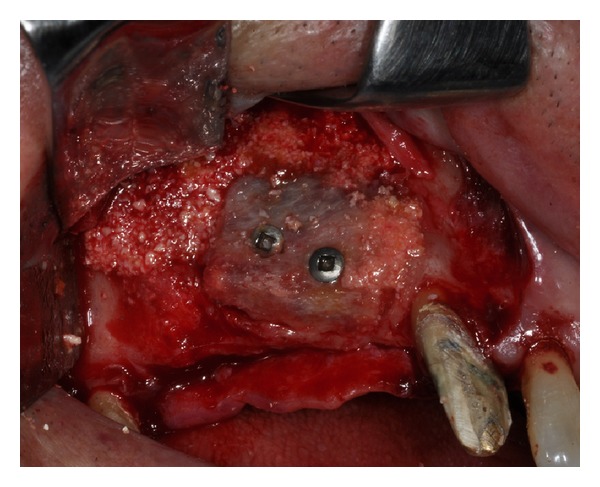
The atrophic upper jaw is treated with a combined sinus lift procedure and local ridge augmentation using fresh frozen homologous grafts.

**Figure 3:**
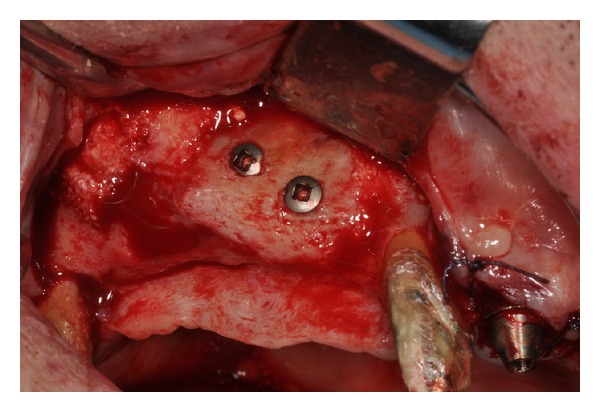
The surgical site is reopened.

Initially, the avulsion of the element 16 was performed and after 40 days, the reconstructive surgery was programmed. On the day of surgery, a sealed container with the graft preserved under controlled temperature was delivered. Once freed from its outer packaging, the specimen was still wrapped in a sealed double sterile bag. It was then transferred to the operating room, where the double bag was opened in a sterile environment and the tissue specimen was defrozen in an abundant solution of saline and rifampicin at a temperature of 37°C for one hour, in compliance with the instructions provided by the reference Bank. Once the specimen had been defrozen, it was debrided to remove nonbony tissue, cut into blocks and contoured or morcellized, based on the treatment plan. The bone deficit was corrected by means of a major sinus lift and an onlay graft using a precontoured allogenic cancellous bone (FFB) block harvested from an iliac crest. The graft was then fixated with osteosynthesis screws. The subantral cavity and the gaps between the graft and the alveolar bone were then filled with allogeneic bone (FFB) chips (Figure 2). The whole thing, including the screws, were then covered with the same morcellized bone, which was maintained in situ by means of resorbable collagen membranes. The wound was closed by sutures after releasing and passivating the flaps. At the clinical controls conducted in the weeks following the surgery and at radiographic examination (OPT) performed 1 month after the surgical procedure, there was no evidence of any complications. Three months later, the avulsions of the elements 12, 11, 21, 22, 24, and 26 were performed, whereas the elements 17, 13, 23, and 27 were maintained in situ and rehabilitated temporarily with a reinforced resin bridge in order to control the vertical dimension, the occlusion and the mandibular movements. At five months, the surgical site was reopened (Figure 3) and the fixation screws were removed. The bone tissue appeared vital and well integrated; no bone resorption was revealed. 

**Figure 4:**
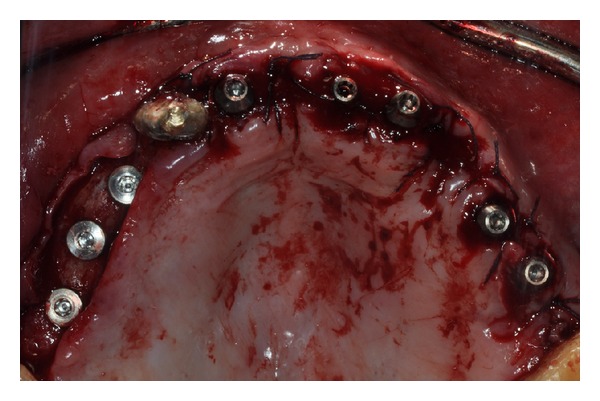
Implant placement.

**Figure 5:**
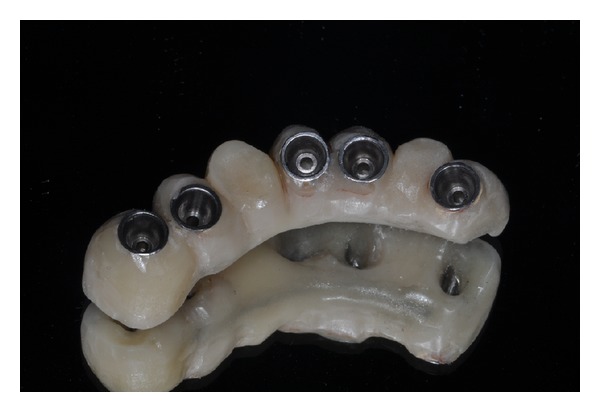
Provisional prosthesis.

**Figure 6:**
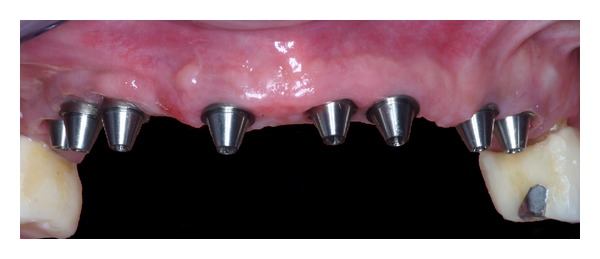
Clinical view. It is possible to appreciate the optimal soft tissue health.

**Figure 7:**
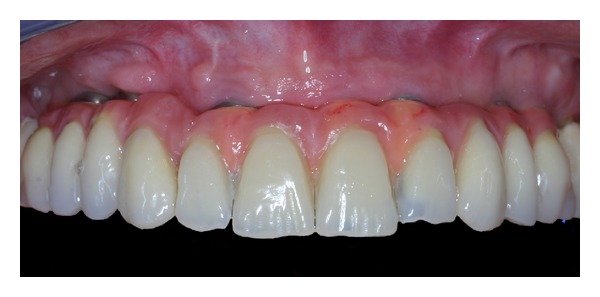
Final screw-retained implant prosthesis.

**Figure 8:**
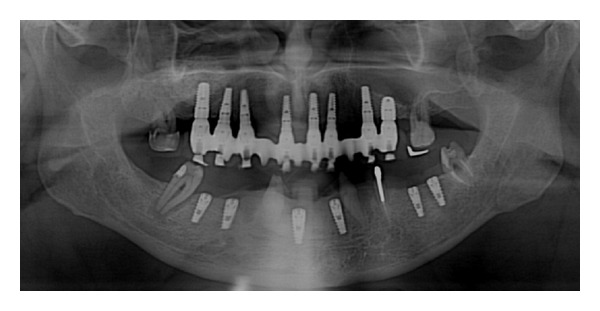
Radiographic followup.

Eight endosseous dental implants (BlueSky, Bredent, Senden, Germany) were inserted at the level of areas 1.6, 1.4, 1.5, 1.2, 2.1, 2.2, 2.4, and 2.5 using a customized surgical template (Figure 4). The dental implants, placed in regenerated bone in the areas 1.6, 1.4, 1.5, were left submerged and loaded at 6 months. Considering the dental implants placed in the incisal region and those located in the areas 2.4 and 2.5, the resulting torque insertion was greater than 35 N/cm. For this reason, immediate loading was performed, and a provisional screw-retained implant prosthesis was fixed on dental implants (Figure 5). Six months after implant placement, a definitive metal ceramic prosthetic rehabilitation was performed (Figures 6, 7, and 8).

**Figure 9:**
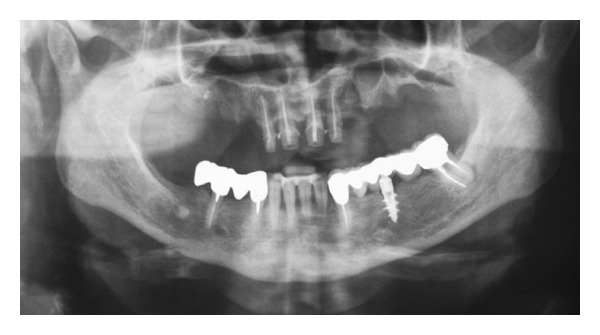
Case 2: initial OPT.

**Figure 10:**
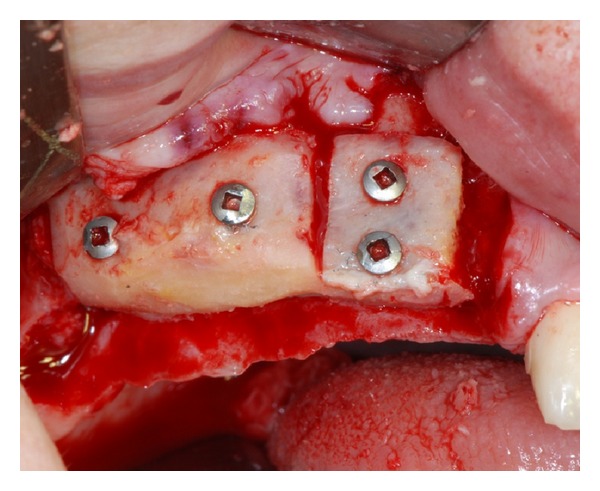
The patient is treated with sinus lift procedure and local ridge augmentation using fresh frozen bone.

**Figure 11:**
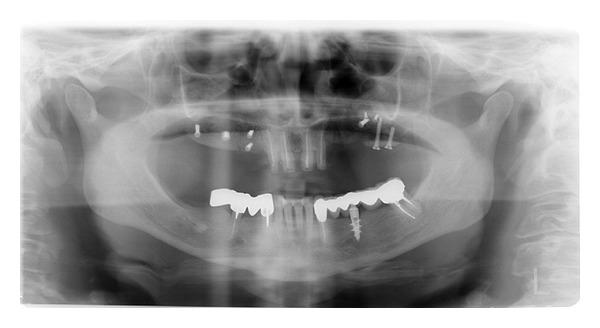
The radiographic image shows that bone grafts are well integrated.

**Figure 12:**
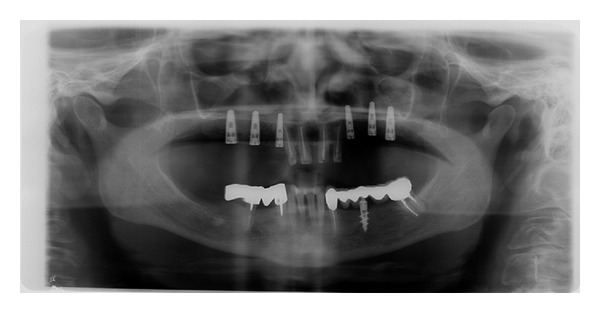
Panoramic radiograph after implant placement.

**Figure 13:**
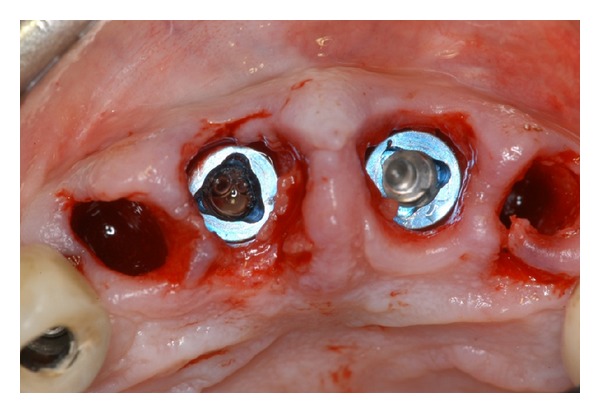
Two postextractive implants are inserted in the incisal area.

**Figure 14:**
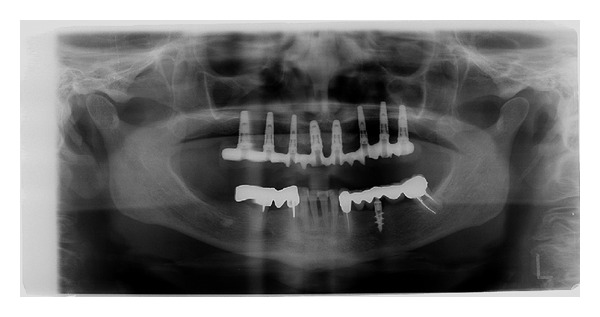
Final X-ray picture.

**Figure 15:**
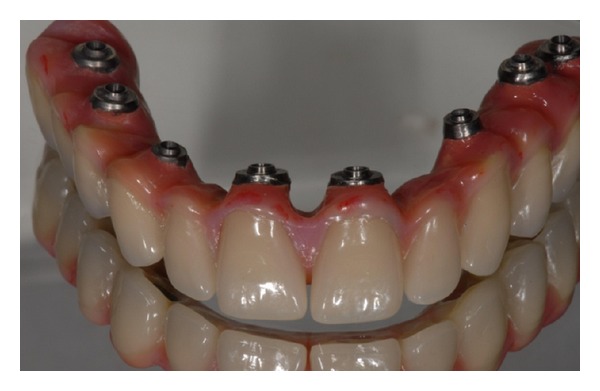
Toronto dental bridge.

*Case 2*. The patient M. P., 60 years old, female required the rehabilitation of the upper jaw because only the incisive teeth were present in the arch. In the posterior regions, the alveolar bone was particularly resorbed (Figure 9). Firstly, the sinus lift procedure and the local ridge augmentation were performed using fresh frozen bone harvested from iliac crest (Figure 10). When the bone graft was well integrated (Figure 11), 6 implants were placed in the posterior areas (NobelReplace, Nobel Biocare, Gothenburg, Sweden) (Figure 12). Five months after implant insertion, the implant sites were reopened, and contextually, the incisive teeth were extracted and postextractive implants were placed in regions 1.1 and 2.1 (Figures 13 and 14). All implants were restored with a Toronto bridge (Figure 15).

## 3. Discussion

When programming a correct treatment plan, the preliminary stages, involving clinical and radiographic examinations, represent the key to determine which treatment strategy should be taken. In particular, the analysis of data acquired by CT with or without the aid of diagnostic and surgical masks, the diagnostic wax-up and the three-dimensional models allow to accurately assess if the quality and quantity of bone are appropriate for achieving high primary dental implant stability, or if simultaneous regenerative techniques are required, or if the implants should be placed after bone reconstructive surgery. In this regard, Buser et al. [20] introduced a classification that relates the bone defect with the possibility of positioning the implants. This classification distinguishes bone defects in 4 classes:

- Class 1: bone volume is ideal both in thickness and height for the correct implant positioning;
- Class 2: moderate horizontal bone resorption does not permit a correct implant axis orientation;
- Class 3: transversal bone resorption. The bone quantity is not sufficient for implant placement, and, for this reason, it is essential to regenerate the bone around fenestrations and dehiscences. In this case, the regenerative techniques are performed in association with implant insertion;
- Class 4: insufficient bone thickness that requires preliminary regenerative techniques and, in a second phase, the performance of implant therapy.

However, when programming a treatment plan, it is important to evaluate the quantitative assessment of bone and to define the implant size and the functional and aesthetic goals of implant rehabilitation. Clinical practice has shown how, in certain cases, it is possible to obviate the preimplant reconstructive surgery using for example, the “short-implants” which have a good behavior in the short and long term as confirmed by the literature [21]. In other cases, however, even in the presence of a sufficient amount of bone for implant insertion, there is no possibility of ensuring the aesthetic and/or functional success of the treatment plan if reconstructive techniques are not performed. In fact, as demonstrated by Dietrich et al. [22] in a 10-year study of 2017 implants, the percentage of implant success is significantly influenced by the thickness of the vestibular bone wall during implant placement. The implant sites that originally had more than 1 mm of vestibular bone wall present a success rate of 96.6% at 5-year followup, while for those implants which originally present less than 1 mm of thickness on the vestibular side, the success rate was 89, 3%. Therefore, the three-dimensional presence of bone around the implants significantly influences the success rates and, if not adequate, determines an aesthetic and/or functional failure of implant rehabilitation. Anyway, nowadays the surgical techniques of preimplant bone reconstruction are highly predictable and are indicated in the presence of vertical and horizontal bone defects.

The autologous bone tissue is considered the gold standard in preimplant bone reconstruction [23]. In fact it ensures the complete absence of adverse immune reaction because the bone is harvested from the patient's own body. In addition, the autologous bone is both osteoconductive, because it provides mechanical support to the vessels and to the cellular elements that will colonize the site of grafting, and osteoinductive because it stimulates osteogenesis [24]. Moreover, since it contains mature cellular elements, the autologous bone has a partial capacity of osteogenesis. However, the autologous bone tissue also presents disadvantages [25, 26]. In fact, the autologous bone graft is indicated in partially edentulous patients because the ridge defects are less severe and more localized, necessitating a smaller quantity of bone. In contrast, an extraoral donor site is often required for bone augmentation where ridge resorption is extreme and extensive. Extraoral bone donor sites provide additional procedural requirements, increased procedure time, and high morbidity and can result in intra- and postoperative complications such as infections of donor or recipient sites.

Considering these issues relative to autologous bone graft, research has been directed towards the study of bone substitutes of various origin (homologous, heterologous, and synthetic). In particular, the homologous fresh frozen (FFB) bone graft has osteoconductive properties and can act as a scaffold by providing structural support during the bone replacement phase. When performed correctly, freezing does not affect the BMPs contained in the bone, so its osteoinductive properties are left unchanged [10, 27]. In consideration of the doubts on the possible interaction between incompatible blood groups and the risk of viral transmission, it must be considered that the assessment of general allogeneic bone donor fitness is more selective than for organ donors and it is based on the collection of in-depth information on the potential donor's medical/social/sexual history, accompanied by a set of instrumental examinations to protect the recipient from transmissible disease [28]. The risk of transmission of viral diseases is now extremely low as PCR serological tests are performed on the donor and then repeated on the tissue during the preparation steps [9]. However, even if the risk of infections is practically absent, the patient should be informed. In addition, the patient must also be informed that he can start donating blood again after control test 90 days after the allogeneic grafting procedure, and the patient can donate all organs with the exception of bone. The use of homologous FFB grafts has some advantages including

- osteoconductive and osteoinductive qualities,
- reduction of postoperative discomfort for the patient due to the lack of donor site,
- availability of graft in suitable quantity and quality,
- limited costs, and
- reduction of operating time because the grafts are prepared on stereolithographic models in a preliminary phase.

The macroscopic clinical results showed in all the clinical cases a successful bone regeneration that allowed the reestablishment of the morphology and bone volume of the alveolar process. Moreover, after five months from graft placement, during the second surgical phase, bone showed a good blood support that indicates the process of bone turnover with new bone formation. The bone regeneration obtained by the FFB graft integration has also been confirmed by several studies that have used homologous FFB bone for reconstructive preimplant surgery. In the clinical cases reported, during implant insertion, a good quality bone was noticed and all implants showed a good primary stability with insertion torque of 35 N/cm. In the literature, studies that refer to the success and survival rates of titanium dental implants placed in regenerated bone using FFB grafts are rather limited. One of the first studies was published in 1992 by Perrott [29] who used FFB graft of iliac crest to rehabilitate, in 8 patients, severe bone atrophy of the jaw. After completing the prosthetic rehabilitation, one implant failed to integrate with regenerated bone, and consequently, the survival rate that resulted was equal to 95.8%. In 2009, Franco [30] evaluated the survival and success rated of implants with narrow diameter (NDI) placed, in regenerated bone with FFB grafts. In this study, 91 ND implants were placed and there were only five failures during a mean observation period of 25 months. The implant survival rate was 95.7%, confirming the results obtained also by Perrott. 

In another study by the same group of authors [9], implants placed in regenerated jaws with FFB grafts were considered. In particular, 21 patients were treated with 28 onlay FFB grafts, and afterwards, 63 titanium dental implants were inserted. During a mean period of 20 months, 2 implants were lost and the survival rate amounted to 96.8%. The studies relating to the implant position in regenerated bone with FFB grafts, even if limited, report encouraging results, similar to those in which the dental implants are positioned in regenerated bone with autologous bone tissue.

For implants placed in native bone and immediately loaded, the osseointegration of all implants was obtained without any complication in the short and long term. The implant-prosthetic techniques of immediate loading are consolidated surgical procedures and are supported by numerous studies. In the literature, in fact, several experimental and histological studies conducted on animal and human models are reported, and these works show that immediate-loaded implants provide promising results compared to delayed-loaded implants. In the clinical cases where the immediate loading is indicated, the immediate implant functionalization allows to obtain a significant reduction of costs and operating time for the patients, and, in particular, if the treatment plan is complex, immediate loading permits a quick restoration of function and esthetics.

## 4. Conclusion

In reconstructive surgery, fresh frozen homologous bone (FFB) allows bone regeneration with its replacement by new bone formation thanks to FFB osteoinductive and osteoconductive properties. In addition, compared to autologous bone, the homologous bone tissue is available in unlimited quantities, allows the reduction of the operating time, and does not have all the disadvantages of the intra- or extraoral surgical site donor.

Therefore, the fresh frozen homologous bone represents a valid alternative to the autologous bone, even for the reconstructions of atrophic jaws. The literature has, in fact, shown that survival rates for implants placed in homologous and autologous bone are comparable with survival rates of over 95% for implants placed in sites regenerated using FFB.
